# Clinical evaluation of semi-automatic landmark-based lesion tracking software for CT-scans

**DOI:** 10.1186/1470-7330-14-6

**Published:** 2014-04-22

**Authors:** Peter Dankerl, Alexander Cavallaro, Matthias Dietzel, Alexey Tsymbal, Martin Kramer, Sascha Seifert, Michael Uder, Matthias Hammon

**Affiliations:** 1 Department of Radiology, University Hospital Erlangen, Maximiliansplatz 1, 91054 Erlangen, Germany; 2 Department of Neuroradiology, University Hospital Erlangen, Maximiliansplatz 1, 91054 Erlangen, Germany; 3 Siemens AG, Corporate Technology, San-Carlos Straße 7, 91054 Erlangen, Germany

**Keywords:** CT, Oncology, RECIST, Lesion tracking, Landmarks

## Abstract

**Background:**

To evaluate a semi-automatic landmark-based lesion tracking software enabling navigation between RECIST lesions in baseline and follow-up CT-scans.

**Methods:**

The software automatically detects 44 stable anatomical landmarks in each thoraco/abdominal/pelvic CT-scan, sets up a patient specific coordinate-system and cross-links the coordinate-systems of consecutive CT-scans. Accuracy of the software was evaluated on 96 RECIST lesions (target- and non-target lesions) in baseline and follow-up CT-scans of 32 oncologic patients (64 CT-scans). Patients had to present at least one thoracic, one abdominal and one pelvic RECIST lesion. Three radiologists determined the deviation between lesions’ centre and the software’s navigation result in consensus.

**Results:**

The initial mean runtime of the system to synchronize baseline and follow-up examinations was 19.4 ± 1.2 seconds, with subsequent navigation to corresponding RECIST lesions facilitating in real-time. Mean vector length of the deviations between lesions’ centre and the semi-automatic navigation result was 10.2 ± 5.1 mm without a substantial systematic error in any direction. Mean deviation in the cranio-caudal dimension was 5.4 ± 4.0 mm, in the lateral dimension 5.2 ± 3.9 mm and in the ventro-dorsal dimension 5.3 ± 4.0 mm.

**Conclusion:**

The investigated software accurately and reliably navigates between lesions in consecutive CT-scans in real-time, potentially accelerating and facilitating cancer staging.

## Background

Radiological staging of cancer patients, especially the time consuming comparison of RECIST (Response Evaluation Criteria in Solid Tumors) lesions in baseline and follow-up CT-scans became a major part of daily radiological routine.

In the era of digital PACS (Picture Achieving And Communication System) and state of the art medical imaging with multidetector computed tomography the amount of image data markedly increased. Several authors addressed the ever increasing image overload, the workflow difficulties of handling huge data volumes and the time-consuming radiological reading and interpretation process [1,2]. Unified standards such as the RECIST-criteria [3] for reading and reporting CTs of oncological patients aim to improve patient care and treatment. However, RECIST-based reporting is known to have the inherent disadvantage of being time-consuming and results show low inter-reader comparability [4].

Previous research that engaged in solving radiological image overload has mainly been directed at improving radiologist’s user interface devices, which enable better navigation in digital data. They conclude that hardware other than the standard mouse, such as a 3D mouse, joystick or infrared controller similar to the Nintendo Wii controller can improve the workflow and optimize image reviewing [5-7]. However, few research has been directed at improving radiological reading software. Modern software could assist radiologists in fulfilling their task of quickly, reliably and comfortably browsing through patient examinations and comparing relevant findings in multiple examinations. Existing radiological software such as in current PACS or 3D image analysis systems feature image synchronisation by picture number, by table position information or by manually assigning corresponding images. These means do not offer a solution as to where in a certain image to look for a finding. The software investigated in this research, is a lesion tracking device, which automatically navigates to lesions in the follow-up CT-examination in real time. The software, however, does not indicate if navigated to lesions change their size in the follow-up examination, instead localizes their anatomical position while leaving the diagnosis up to the radiologist.

The aim of this study was to evaluate the feasibility, accuracy and reliability of an automatic landmark-based lesion tracking software enabling navigation between RECIST-lesions in baseline and follow-up CT-scans.

## Methods

Institutional Review board approval was obtained for this study and all procedures were in accordance with the Helsinki Declaration.

### Patient selection and scan technique

We searched the Radiological Information System (RIS) for consecutive oncologic patients that presented at least one lesion in the thorax, one lesion in the abdomen and one lesion in the pelvis and that received at least two computed tomography (CT)-scans within 6 months. 32 patients (26x lymphoma, 4x melanoma, 2x prostate cancer; 21 males 11 females; mean age 51.2 ± 15.3 years, range 21 to 76) were enrolled for experimental analysis. Two consecutive CT-scans were read according to the RECIST guidelines.

CT-examinations were indicated by clinical needs in all cases. Patients were examined with a 64-row multidetector CT-scanner (Somatom 64, Siemens AG, Erlangen, Germany). The tube voltage was 120 kV, the pitch was 0.75 and the collimation was 0.6 mm. Further, the mean CTDIvol value in investigated scans was 11.2 ± 2.4 (range 6.7 – 16.3). A weight-dependent dose of 350 mg per ml iodine i.v. contrast agent (Imeron 350®, Bracco Imaging, Konstanz, Germany) was administered to all patients with a flow-rate of 3 ml/sec.. Images were acquired in the portal-venous contrast agent phase. For every patient a data-set with 1 mm slice thickness and a soft tissue kernel was calculated. If clinically indicated contrast agent was administered orally or rectally in accordance with guidelines (1.5 liters of water containing 60 ml of Gastrografin®, Bayer, Leverkusen, Germany). All CT-scans were performed in the Department of Radiology of the University Hospital Erlangen.

### Lesion tracking software

The software was developed within the scope of the Theseus-Medico research project. This is a 5-year non-commercial, nationwide multi-centre research project started in 2007, with physicians, university health care professionals, technicians and computer scientists as joint venture.

**Figure 1:**
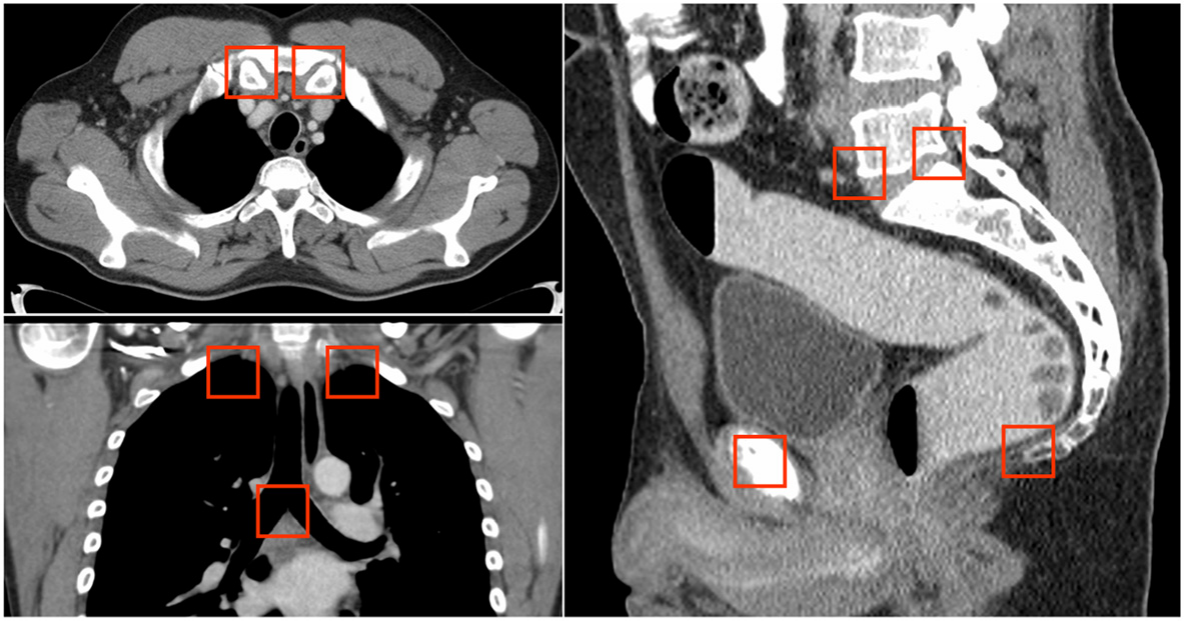
**Detected CT landmarks.** Examples of automatically detected anatomical landmarks in a thoraco/abdominal/pelvic CT-scan (inner tips of left and right clavicle, top of right and left lung, bronchial bifurcation, front and back of bottom plate of lumbar vertebra 5, pubic symphysis and coccygeal bone). Automatic detection of 44 thoracic, abdominal and pelvic landmarks in each CT-scan enables setting up a patient specific coordinate-system, cross-linkage of the coordinate-systems of consecutive CT-scans and a software-based navigation between baseline and follow-up CT-scans.

The lesion tracking software features a novel pattern recognition method based on the principles of machine learning [8] which enables automated detection of stable anatomical landmarks. Landmarks are hereby defined as three dimensional points in CT-scans, for instance the inner tip of left and right clavicle, the dorsal tip of left and right spina scapulae, the top of both lungs, the bronchial bifurcation, the front and back of base plate of lumbar vertebra 5, etc. (Figure 1). Their detection is supposed to be independent of contrast enhancement and contrast agent phase (native, portal venous, arterial, etc.) [8]. In total, 44 landmarks are automatically detected in every thoraco/abdominal/pelvic CT-scan in each patient. To enable robust and quick landmark detection, anatomic/geographical information, such as “to the right of”, “close to”, “above”, etc. is computed with the position of detected landmarks and supports the localization of further landmarks [8]. Then a patient specific graphical network between all detected landmarks is set up for each CT-scan. Synchronizing the graphical networks from the baseline and follow-up CT-scan of one patient enables the computer-aided-navigation [8]. Absence of certain landmarks in a given CT-scan, for example if only an abdominal scan was performed, is notified as well. This facilitates the software to recognise which body region was scanned [9]. The lesion tracking software facilitates side-by-side comparison of consecutive examinations by indicating corresponding findings with a crosshair. Radiologist can automatically navigate to corresponding locations in the follow-up examinations by clicking on a lesion in the baseline-study, all in real time. The crosshair synchronization is accomplished by an elastic image registration method based on corresponding anatomical landmarks and approximating by thin-plate splines [8]. For this purpose, the detected landmarks from the currently considered images are related to the manually annotated landmarks of an atlas. The investigated application further utilizes the mathematical algorithms “Marginal Space Learning”, “Probabilistic Boosting Tree” and “3D Haar features”. Technical and mathematical (algorithm) details are described more detailed in the Additional file 1.

**Figure 2:**
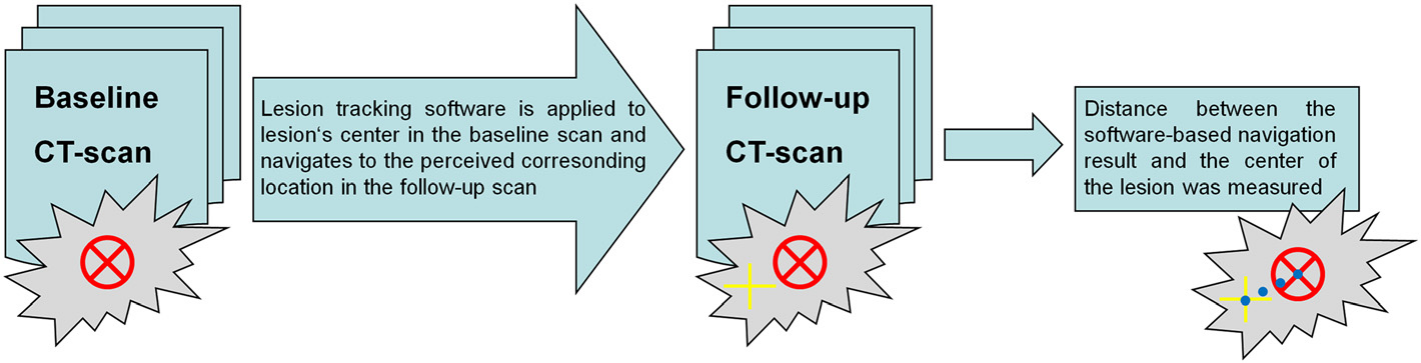
**Experimental setup.** In a consensus assessment of three readers the centre of each RECIST target and non-target lesion was marked in every baseline and follow-up CT-scan (red X). Next, the readers applied the lesion-tracking software by clicking on each defined centre of the lesions in the baseline CT-scans. Thus, the software automatically navigated to the predicted corresponding location in the follow-up CT-scan (yellow crosshair). The deviation between lesion’s centre and the navigation result of the software was documented as distance (blue dotted line) in all three special dimensions (cranio-caudal, ventro-dorsal, lateral).

### Experimental setup and evaluation

To determine the accuracy of the lesion tracking software we evaluated the deviation between the centre of each lesion (reference standard) and the location the automated lesion tracking software navigated to in the follow-up examination. The centre of 96 RECIST target and non-target lesions (such as metastasized lymph nodes or metastases of the lung or parenchymal abdominal organs) was marked in every baseline and follow-up CT-scan. In a consensus assessment of three readers (with 16, 4 and 3 years of work experience) the centre of each lesion and the deviation to the navigation result of the software was documented (Figure 2). To increase the power and validity of our study we investigated various individuals and independent lesions in different body regions with continuous instead of categorized measurement [10]).

The lesion tracking software was evaluated on a standard computer (Dual Core Xeon 2.66 GHz; Windows XP, 32 Bit) and dedicated diagnostic displays (Coronis Fusion 6MP LED, Barco, Kortrijk, Belgium).

### Statistical analysis

System’s runtime was computed and mean runtime ± standard deviation was calculated.

Mean deviation ± standard deviation was calculated for every spatial direction (cranial, caudal, ventral, dorsal, lateral-left and lateral-right). To investigate a possible systematic error differences between mean deviations were calculated. Furthermore the unsigned mean deviation ± standard deviation for each of the three spatial dimensions (cranio-caudal, ventro-dorsal and lateral) was calculated and therefrom mean vector length ± standard deviation of deviations between lesions’ centre and the semi-automatic navigation result was calculated.

## Results

The mean runtime the lesion tracking software required to initially synchronize baseline and follow-up examinations was 19.4 ± 1.2 seconds. Thereafter, the navigation between corresponding RECIST lesions in baseline and follow-up examinations was performed in real-time.

**Figure 3:**
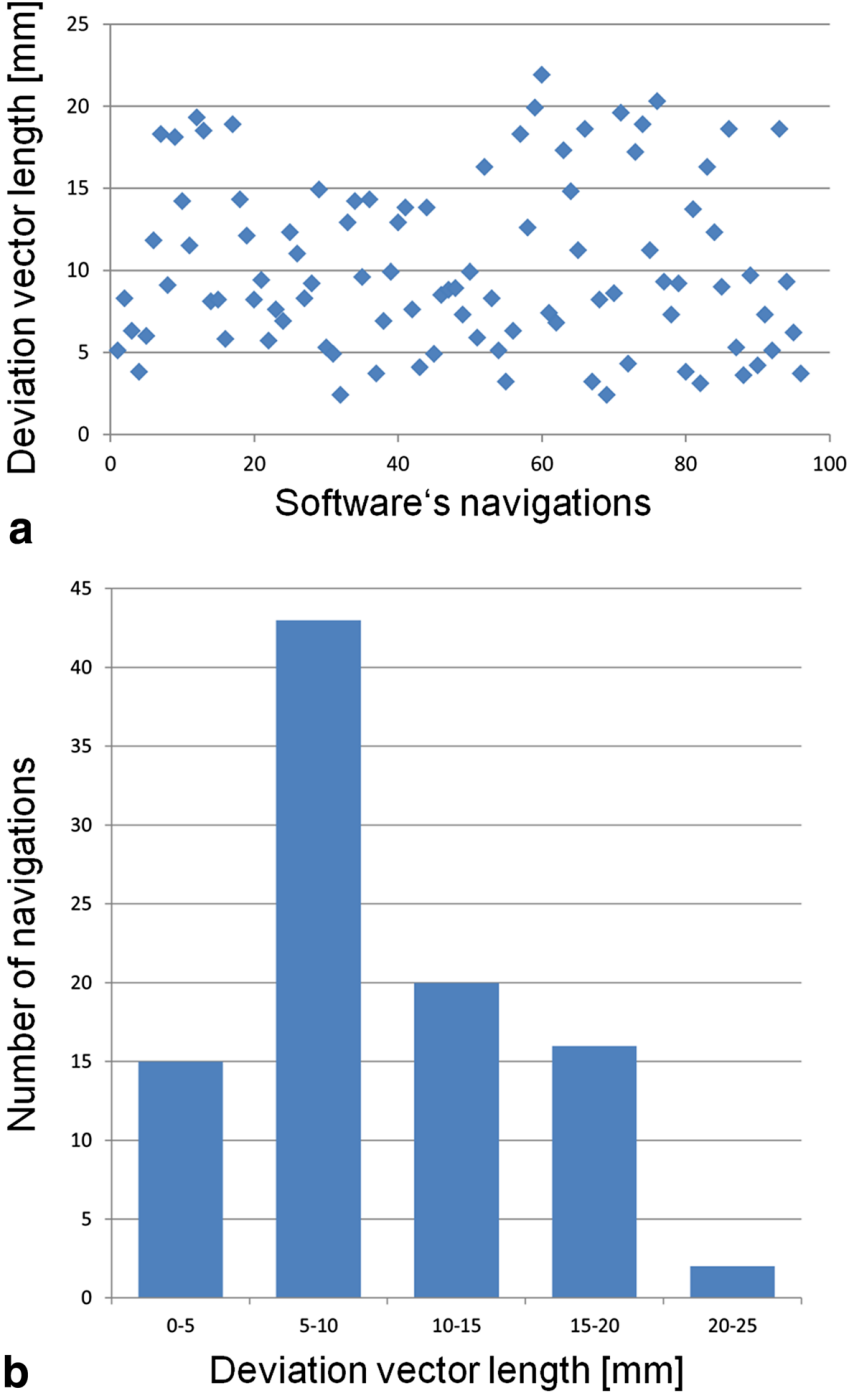
**Deviation vectors.** Graphical display of deviation vector length between the center of 96 RECIST target and non-target lesions (reference standard) and the locations the automated lesion tracking software navigated to in the follow-up examination. **a)** presents the deviation vector lengths as a scatter plot; **b)** as a histogram.

Mean deviations were the following: 5.1 ± 4.1 mm (range 1.0 – 14.7) lateral-right, 5.2 ± 3.7 mm (range 0.7 – 14.2) lateral-left, 5.5 ± 4.2 mm (range 0.8 – 14.3) ventral, 5.1 ± 3.8 mm (range 0.7 – 13.8) dorsal, 4.7 ± 3.4 mm (range 0.8 – 12.8) cranial and 5.9 ± 4.3 mm (range 1.2 – 11.9) caudal (Table 1). Figure 3 depicts deviation vector lengths between the reference standard and the location the automated lesion tracking software navigated to in the follow-up examination. Differences between mean deviations within the dimensions were 0.1 mm toward lateral left, 0.4 mm toward ventral and 1.2 mm toward caudal.

**Table 1:** Mean runtime (± standard deviation) the lesion tracking software required to initially synchronize baseline and follow-up CT-scans is shown in seconds

**Software’s initial runtime (sec)**	19.4 ± 1.2
**Deviation (mm)**	
Lateral-right	5.1 ± 4.1 (1.0 – 14.7)
Lateral-left	5.2 ± 3.7 (0.7 – 14.2)
Ventral	5.5 ± 4.2 (0.8 – 14.3)
Dorsal	5.1 ± 3.8 (0.7 – 13.8)
Cranial	4.7 ± 3.4 (0.8 – 12.8)
Caudal	5.9 ± 4.3 (1.2 – 11.9)
Lateral dimension	5.2 ± 3.9 (0.7 – 14.7)
Ventro-dorsal dimension	5.3 ± 4.0 (0.7 – 14.3)
Cranio-caudal dimension	5.4 ± 4.0 (0.8 – 12.8)
**Mean length of the deviation vector (mm)**	10.2 ± 5.1 (2.4 – 21.9)

Mean distances ± standard deviations between the software-based navigation results and the centre of the lesions in follow-up CT-scans is shown for all directions and dimensions. Mean length ± standard deviation of the deviation vector was calculated.

Range is given in brackets.

The calculated unsigned mean deviation was 5.4 ± 4.0 mm (range 0.8 – 12.8) in the cranio-caudal dimension, 5.2 ± 3.9 mm (range 0.7 – 14.7) in the lateral dimension and 5.3 ± 4.0 mm (range 0.7 – 14.3) in the ventro-dorsal dimension. This resulted in a mean length of the deviation vector of 10.2 ± 5.1 mm (range 2.4 – 21.9).

**Figure 4:**
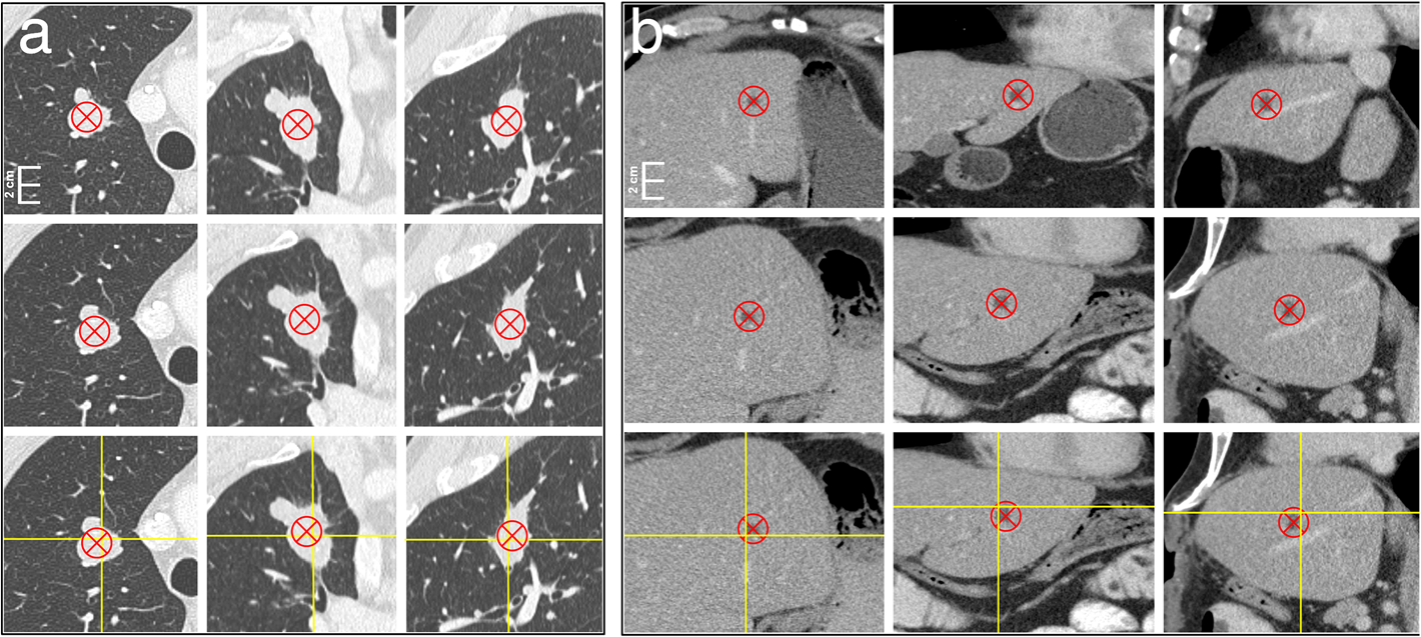
**Examples. a)** An example navigation result of the lesion tracking software applied to a thoracic lesion (lung metastasis). **b)** An example navigation result of the lesion tracking software applied to an abdominal lesion (liver metastasis). First row shows the manually marked centre of a target lesion in the baseline CT-scan (red X = reference standard; axial, coronal and sagittal view). Second row shows the manually marked centre of a target lesion in the follow-up CT-scan of the same patient (red X = reference standard; axial, coronal and sagittal view). Third row shows the follow-up CT-scan with the software-based navigation result (yellow crosshair) and the reference standard (red X).

Examples of lesion tracking software’s results are demonstrated for a lung metastasis (RECIST target lesion) and for a liver metastasis (RECIST non-target lesion) (Figure 4).

## Discussion

The investigated lesion tracking software demonstrated a deviation vector of roughly 1 cm length on average, when semi-automatically navigating from lesions in the baseline CT-examinations to the corresponding locations in follow-up scans. The average deviation vector composes of divergences in all three spatial directions. It is the three-dimensional distance between the location the software automatically navigated to in the follow-up scan and the reference standard. This does not correspond to an absolute two-dimensional deviation (e.g. in the axial plain) of 10 mm – in fact the average two-dimensional deviation error (deviations in each direction) in our study was never greater than 5.9 mm. We hold this deviation to be clinically tolerable as lesions which are to be classified as RECIST-target lesions (which have to be compared in baseline and follow-up CT-scans) have to be of a certain size. Lung nodules or liver lesions have to be 10 mm in largest diameter, while lymph nodes even have to measure 15 mm in the short axis (RECIST 1.1 [11]). As no single of our spatial deviations was greater than 5.9 mm, we conclude that navigation to target lesions with the presented software is very accurate and of clinically tolerable error.

The semi-automatic navigation between corresponding lesions in consecutive CT-examinations presumably saves time during tumor response evaluation. Moreover, only few navigational results demonstrated larger (of about 15 mm) deviation errors. In these cases, the lesions were located in or close to the abdominal wall and thereby relatively far away from the majority of software detected landmarks (mostly located in the spine and the pelvis). This deviation error can even be reduced when more landmarks are implemented into the system. Another possibility for deviational errors might be an uncertain reference point of the lesions in baseline and follow-up due to changes in lesion dimension after chemotherapy. In order to minimize this error, 3 radiological readers determined the reference points in consensus. If certain landmarks cannot be detected (e.g. in osteochondrosis between lumbar vertebra 5 and sacral vertebra 1, or scoliosis) within a certain computational time this landmark is ignored by the algorithm and the graphical network is constructed with the remaining landmarks. Thereby the software system remains stable and functioning even if it cannot detect all landmarks. In our patients the software did not fail to detect landmarks.

To investigate the clinical feasibility of this software, we examined the software utilizing consecutive routine clinical CT-scans, as requested by van Ginneken [12]. The only inclusion criterion was that patients show at least one lesion in the thorax, in the abdomen and in the pelvis to prove accuracy of the software in different body regions. The software apprehended all CT-examinations and no scan was excluded due to software inability. Therefore, the presented lesion tracking software proofed to be feasible and reliable in clinical routine CT-scans.

After the initial synchronization of the data sets, which takes about 20 seconds and can be performed as a preprocessing step, subsequent navigation between lesions in different CT-scans is realized in real time. Therefore, the software facilitates handling of large data sets without delay. Andriole et al. address the increasing image overload and the thereby resulting increased workload for radiologists [1,2]. However they do not offer a solution from a software standpoint. Addressing this issue, usage of a robust lesion tracking tool is an important point.

The presented software tool is unique in various ways. It automatically detects stable anatomical landmarks throughout the body and sets up a patient specific graphical network between the landmarks, comparable to the global positioning system (GPS) navigation. Beside navigation in matching CT-scans, GPS-like navigation potentially enables comparing findings in non-matching body regions (e.g. comparing a thoracic with a thoraco/abdominal CT-scan). At present, commercial tools by various vendors, for instance for the follow-up of lung lesions are available. However, these tools mainly rely on deforming of images based upon Hounsfield Unit (HU) similarities. According to Seifert et al. [8] this strategy has the drawback that changes in organ size, e.g. splenomegaly, or topographical changes like nephrectomy cannot be apprehended. Moreover, synchronous navigation to findings in organs of non-matching body areas is a big obstacle for such software [8]. As an example a liver lesion included in a baseline thoracic/upper abdomen CT-scan and a follow-up abdominal/pelvic CT-scan cannot be synchronized by HU similarities or table position. Presented landmark based navigation software possibly overcomes these limitations due to its underlying algorithm [8].

Another approach for navigation assistive software is to just align axial CT-slices according to picture number. Here different patient positioning on the CT-table and different scanning start points result in poor matches. Further these pieces of software do not indicate which explicit finding on an axial slice was evaluated in the previous examination if there are multiple lesions on the same slice. In contrast, the evaluated software navigates to a certain finding and specifically indicates it by placing a crosshair on the corresponding finding. Finally, the landmark-based approach requires less computational power and consequently less run-time than systems relying on matching HU similarities [8].

CAD systems have two common goals, to improve accuracy and increase radiologists’ productivity [13]. Recently, various computer-aided detection (CAD) systems for multiple clinical tasks have been developed, such as CAD for lung cancer [14], pulmonary embolism [15], breast cancer [16], colon cancer [17], liver cancer [18], prostate cancer [19] and coronary artery stenoses [20]. Due to its landmark based framework the software fulfills two of Kauczor’s demands for a CAD system [21]: It generates a 3D map and registrates different imaging series facilitating orientation and navigation in CT-scans.

In order to compare consecutive oncological measurements the same, and if possible thinnest slice thickness should be used [22]. This calls for radiological reading with a 3D radiological image analysis system (e.g. MicroView® (Parallax Innovations, Ilderton, Canada), Aquarius® (TeraRecon, Foster City, CA, USA) or SyngoVia® (Siemens AG, Erlangen, Germany)). These, however do not feature image numbers. This even complicates the evaluation of findings in baseline and follow-up studies when having to navigate manually between lesions (e.g. by scrolling through the examinations). For this workflow an additional automatic or semi-automatic navigation solution seems even more needed than with conventional PACS reading (e.g. reconstructed 5 mm slice thickness data sets).

Our study faces some limitations that suggest directions for future work. One limitation was that the software’s proposed time saving effect for oncological reading was not assessed. This is owed to the fact that the aim of our research was to evaluate the clinical feasibility, accuracy and reliability of the software.

Another limitation was that in this study we concentrated on navigation within full-body (thoraco/abdominal/pelvic) CT-scans. However, we did not investigate the technically possible [8] navigation between non-matching body regions.

One further plan is to combine the features of presented lesion tracking software with features of automatic lesion volumetry software.

## Conclusions

The presented and evaluated lesion tracking software reliably handled clinical routine CT-scans and accurately enabled semi-automated real-time lesion tracking with a clinically tolerable accuracy. The software presumably facilitates the handling of large CT data sets, eases the visualization and evaluation of tumor response in consecutive CT-scans, and potentially speeds up radiological reading.

## Consent

Institutional review board approval was obtained for this study. The need for informed consent was waived. All procedures were in accordance with the Helsinki Declaration.

## Competing interests

This research was done within the scope of the German governmental funded Theseus-Medico project (January 2007 – December 2012), a non-commercial, nationwide multi-centre research project including physicians, university healthcare professionals, computer scientists and collaborators from industry. Further detail concerning Theseus-Medico can be accessed via: http://theseus.pt-dlr.de/en/920.php.

There is no conflict of interest for the authors from the Siemens AG (AT, MK, SS), or the authors from the Department of Radiology from the University Hospital Erlangen (PD, AC, MD, MU, MH).

## Authors’ contributions

Authors PD, AC, MH conducted the experiments; authors AT, MK, SS developed the software; authors PD, MD, AT, MH wrote the manuscript and did the statistical analysis; authors AC, MU revised and edited the manuscript. All authors read and approved the final manuscript.

## Supplementary Material

Additional file 1 Technical and mathematical algorithm details Click here for file
