# Supplementary material for: Clinical evaluation of semi-automatic landmark-based lesion tracking software for CT-scans
Source: Cancer Imaging. 2014 Apr 22;14(1):6. doi: 10.1186/1470-7330-14-6 (PMC4212533; doi:10.1186/1470-7330-14-6)
Supplement: Additional file 1 — Technical and mathematical algorithm details [file 1470-7330-14-6-S1.doc]

**Additional file 1**

**Technical Method**

Intrinsic Random Forest similarity

For a Random Forest (RF) trained for a certain classification problem, the proportion of the trees where two instances appear together in the same leaves can be used as a measure of similarity between them [1]. For a given forest ***f*** the similarity between two instances ***xi*** and ***xj*** is calculated as follows. The instances are propagated down all ***K*** trees within f and their terminal positions ***z*** in each of the trees ***(zi = (zi1, , ziK)*** for ***xi***, similarly ***zj*** for ***xj***) are recorded. The similarity between the two instances then equals to:

where I is the indicator function. When dissimilarity or distance is needed and not a similarity (e.g., for clustering or multi-dimensional scaling) it is normally calculated as suggested by Breiman [1]:

The intrinsic RF dissimilarity is known not to be metric [2], as the triangular inequality is often violated:

Several reasons motivate the choice of learning algorithm in our framework. First, RF was demonstrated to work well with and be robust to high-dimensional data with many weakly relevant, redundant and noisy features, without the need for additional data pre-processing and feature selection. Next, RF-based models are relatively fast to train and to apply comparing for example with Support Vector Machines. Then, RFs can be trained both for classification and regression problems, support supervised learning from multiple categories, and can easily handle missing values. Last but not least, they are able to provide the intrinsic RF similarity, which helps to combine the discriminative power and robustness of RFs with the transparency of case retrieval and nearest neighbor classification or regression. Thanks to the appealing properties of RF, the RF similarity can be easily calculated for different tasks, including classification and regression problems, tasks with heterogeneous feature vectors with possible missing values, and multiclass tasks.

In order to speed up our extensive experiments, make it possible to validate more trends and make the models adaptive to learn from new samples we implement an incrementalization of RF similar to Saffari [3]. While successful lossless incrementalizations exist for many learning algorithms, most strong techniques applied in real applications are still difficult to incrementalize, and among them perhaps the most prominent example that received considerable attention lately is the ensemble of randomized decision trees, the most famous representative of which is the Random Forest algorithm [1]. A few algorithms for online ensembles of randomized decision trees have been recently proposed, and their application to vision tasks (in particular tracking) have been considered, despite the fact that they are not lossless and often require considerably more training cases than the corresponding batch technique in order to converge.

In particular, the online RF of Saffari et al. [3] trains decision trees of fixed depth and has a fixed structure which does not change with the observation of new cases once the tree depth limit is reached. We address this issue in our online RF algorithm with

- The use of primed off-line learning to speed up convergence to a reasonable accuracy.
- Different sources of randomness (including bagging modeled with Poisson distribution of instance weights, and a random sample of observed features at each node).
- Memory management to avoid exceeding a specified memory limit for the model.
- Restructuring of the trees according to observed changes in the data distribution.

The training process is started with the classical Random Forest algorithm [1] with 100 trees. Each decision tree is subsequently refined using online training. Online learning allows the framework to be scalable to the number of training instances. For each tree, a random sample of 200 lesions with even class distribution is used for the primed training. Primed off-line training is a simple but effective technique to improve the predictive performance of the final model (see [4] for an example).

We exploit the memory management scheme proposed before for online Hoeffding trees that dynamically activates most promising nodes, for tracking feature distributions and a split attempt, and deactivates and removes the less promising ones [5]. Similar to Saffari et al. [3] and different to the Hoeffding tree, a split is simply generated after observing a certain specified number of instances (40 is the default value normally leading to best performance). For each feature, Gaussian distribution is assumed and is tracked online, and a split threshold value which maximizes the *Gini Index* value is selected.

Liver lesion texture descriptors

Each liver lesion in our experimental setting is described by a set of low–level computer–generated imaging features as follows:

- Relative frequency histogram and four first central normalized moments on it for Hounsfield Units (HU) distribution in the bounding box for the lesion.
- The set of eight invariant Hu moments of order up to 3 [6].
- Six invariant Zernike moments [7].
- HU histogram and the four first central moments for the whole liver.

The first three feature types describe the lesion itself, while the last one describes the image of the whole liver. Inclusion of whole liver features has been shown to always lead to an improved discrimination performance in our set of experiments. We use 2D Hu and Zernike invariant moments [6, 7]. In order to adapt them to the 3D liver lesion, for each lesion we generate 3 orthogonal 2D cuts intersecting at the centre of the lesion ROI. The invariant moments are then calculated for each cut, and the feature vector includes both the moment for each separate cut and the averaged moments.

**Appendix References**

1. Breiman L. Random forests. Machine Learning 2001; 45, 5–32.
2. Tsymbal A., Huber M., Zhou S. Learning discriminative distance functions for case retrieval and decision support. Transactions on CBR 2010; 3(1), 1–16.
3. Saffari A, Leistner C, Santner J, et al. On-line random forests. 3rd IEEE ICCV Workshop on Online Computer Vision 2009; 1393-1400.
4. Oza NC, Russell S. Experimental comparisons of online and batch versions of bagging and boosting. Proceedings of the seventh ACM SIGKDD international conference on Knowledge discovery and data mining 2001; 359-364.
5. Pfahringer B, Holmes G, Kirkby R. New options for hoeffding trees. AI: Advances in Artificial Intelligence. Lecture Notes in Computer Science Volume 2007; 4830, 90-99.
6. Hu MK. Visual pattern recognition by moment invariants. IRE Trans. Inform. Theory 1962; 8, 179-187.
7. Pejnovic P, Buturovic L, Stojiljkovic Z. Object recognition by invariants. Proceedings of Int. Conf. on Pattern Recognition 1992; 434-437.
